# A Flow Cytometry-Based FRET Assay to Identify and Analyse Protein-Protein Interactions in Living Cells

**DOI:** 10.1371/journal.pone.0009344

**Published:** 2010-02-22

**Authors:** Carina Banning, Jörg Votteler, Dirk Hoffmann, Herwig Koppensteiner, Martin Warmer, Rudolph Reimer, Frank Kirchhoff, Ulrich Schubert, Joachim Hauber, Michael Schindler

**Affiliations:** 1 Heinrich-Pette-Institute for Experimental Virology and Immunology, Hamburg, Germany; 2 Institute of Clinical and Molecular Virology, University of Erlangen-Nuremberg, Erlangen, Germany; 3 Institute of Virology, University of Ulm, Ulm, Germany; Institut Pasteur Korea, Republic of Korea

## Abstract

**Background:**

Försters resonance energy transfer (FRET) microscopy is widely used for the analysis of protein interactions in intact cells. However, FRET microscopy is technically challenging and does not allow assessing interactions in large cell numbers. To overcome these limitations we developed a flow cytometry-based FRET assay and analysed interactions of human and simian immunodeficiency virus (HIV and SIV) Nef and Vpu proteins with cellular factors, as well as HIV Rev multimer-formation.

**Results:**

Amongst others, we characterize the interaction of Vpu with CD317 (also termed Bst-2 or tetherin), a host restriction factor that inhibits HIV release from infected cells and demonstrate that the direct binding of both is mediated by the Vpu membrane-spanning region. Furthermore, we adapted our assay to allow the identification of novel protein interaction partners in a high-throughput format.

**Conclusion:**

The presented combination of FRET and FACS offers the precious possibility to discover and define protein interactions in living cells and is expected to contribute to the identification of novel therapeutic targets for treatment of human diseases.

## Introduction

One of the few non-invasive techniques to study protein interactions is Försters resonance energy transfer (FRET) [1], [2]. FRET is based upon the transfer of energy from an excited donor fluorophor to a close-by acceptor fluorophor, resulting in enhanced fluorescence emission of the acceptor [3]. This phenomenon only occurs when the distance between donor and acceptor is less than 10 nm and the emission spectra of the donor overlaps with the excitation of the acceptor [3]. While FRET-methods have been improved in the last years [4], [5], major limitations still exist. Due to the spectral overlap between donor and acceptor it is difficult to get a clear FRET signal and extensive controls and complicated software calculations are needed to eliminate artefacts [6], [7]. Other FRET approaches that are less artefact prone, such as fluorescence lifetime imaging (FLIM), require special equipment and expert knowledge [8]. Most importantly, FRET measurements are generally done by fluorescence microscopy, which is tedious and essentially precludes the analysis of large cell numbers as well as high-throughput-screening (HTS) for protein interactions [1], [2].

One possibility to overcome these limitations is to detect and quantify FRET signals by flow cytometry. Fluorescence activated cell sorting (FACS) is non-invasive, sensitive and quantitative and allows to measure large numbers of cells and samples in a reasonable amount of time [9]. Thus, FACS-based FRET could be well suited to study protein interactions in living cells. Surprisingly, this technology was so far only applied to a few special scientific questions [9], [10], [11], [12], [13]. The reason for this might be that an easy to adapt, standardized, well controlled and reliable routine to measure and quantify FRET by FACS is still missing. Our goal was to establish a versatile FACS-based FRET assay using the standard FRET pair CFP/YFP [14]. We evaluated this methodology by investigating interactions between the human and simian immunodeficiency virus (HIV and SIV) Nef and Vpu proteins and various cellular factors [15], as well as HIV Rev multimerization [16]. Furthermore, we demonstrate that HIV and SIV Nef bind to the primary viral receptor CD4 with comparable efficiency. In contrast to this, SIV Nef interacts with CD3 to a much higher extent as Nef of HIV-1 does. Additionally, we show direct binding of HIV-1 Vpu to CD4 and the recently described restriction factor CD317 (also termed Bst-2 or tetherin), which inhibits retroviral particle release from infected cells [17]. Mutation of amino acid residues in the membrane spanning region of Vpu specifically diminished its capacity to bind CD317. Finally, we demonstrate the applicability of our assay for HTS by successful sorting of FRET positive cells and subsequent plasmid isolation. The established method overcomes current limitations in proteomics, allowing scientists to identify and analyse protein interactions in any compartment of living mammalian cells.

## Materials and Methods

### Generation and Cloning of Expression Vectors

Our aim was to develop a cloning strategy that allows to generate any gene of interest (GOI) as an N- or C-terminal EYFP/ECFP-fusion without further modifications of the vectors (Fig. S1). Therefore, we used the widely distributed Clontech vectors pEYFP-C1/N1 and pECFP-C1/N1 (kind gifts from Dr. Klaudia Giehl, University of Ulm). In C1- and N1-vector derivatives C-terminal tagged fusions can be generated by using the single cutter restriction sites *NheI* and *AgeI*. Target sequence amplification is done with 5′-p*NheI*-GCG*GCTAGC*-(target sequence) and 3′-p*AgeI*-TCG*ACCGGT*GCACCTGCTCC-(target sequence) which eliminates the stop codon and introduces the linker AA sequence GAGAPVAT. Similarly, N-terminal tagged fusions in C1-vector derivatives can be generated by using the unique *XhoI* and *EcoRI* sites and amplification of the target with 5′p*XhoI*-CG*CTCGAG*CT-(target sequence) and 3′-*EcoRI*-CT*GAATTC*-(target sequence) resulting in the linker SGLRSRA. In N1-vector derivatives N-terminal tagged fusions are generated via the *BsrGI* and *NotI* sites and the primer 5′p*BsrG1*-GC*TGTACA*AGGGAGCAGGTGCAGGAGCA-(target sequence) and 3′p*NotI*-GTC*GCGGCCGC*T-(target sequence) resulting in the linker LYKGAGAGA. An overview of the vectors and restriction sites used, as well as the linker sites is depicted in Figure S1. The membrane expressed pECFP-MEM (Clontech) control was a kind gift of Dr. Klaudia Giehl (University of Ulm). As FRET-positive control we generated the pEYFP-ECFP construct expressing EYFP and ECFP as a fusion. Cellular factors (MHC-I, MHC-II, CD3ζ-chain, CD4, CD317, MurrI and p53) were PCR amplified from a human PCR-ready PBMC cDNA (Spring Bioscience) and ligated into pECFP-C1 using standard cloning procedures. All factors were generated as C-terminal tagged ECFP fusions except CD317, which was tagged at the N-terminus. HIV-1 NL4-3 *vpu, tat* and NA7 *nef* as well as SIVmac 239 *nef* were PCR amplified from proviral DNA and ligated into pEYFP-N1 or pEYFP-C1 as C-terminal tagged fusions. CD317 was PCR amplified and inserted into pFLAG-CMV2 (Sigma) that directs the expression of CD317 N-terminally tagged with a FLAG epitope. HIV-1 C-terminal tagged Rev-fusion constructs were amplified by PCR from HIV-1 pcRevWT and pcRevSLT40 [16] and ligated into pEYFP-N1 or pECFP-N1. For demonstration of HTS, we PCR amplified the pEYFP-*vpu* fusion and inserted it into the pCGCG-vector [18] instead of the IRES-GFP cassette. All PCR derived inserts were sequenced to verify the absence of undesired nucleotide changes.

### Cell Culture and Transfections

293T or Hela cells were maintained in Dulbecco modified Eagle medium (DMEM) supplemented with 10% FCS. 293T cells were transfected by the calcium phosphate method as described previously [18]. Briefly, 400,000 cells/well were seeded in 6-well plates one day prior to transfection. Then we transfected 2.5 µg DNA per donor and acceptor construct and FRET measurements were performed 24–36 h post transfection. For the Rev multimerization experiments 293T cells were transiently transfected with 0.5 µg Gag expression vector GPV-RRE [19] (provided by M.H. Malim; King's College London, UK), 0.125 µg pBC12/CMV/SEAP (transfection-efficiency control) and 0.25 µg of acceptor and donor construct by using TurboFect reagent (Fermentas) according to the manufactor's protocol. Additionally to FRET measurements supernatants were analysed for particle production by p24 antigen ELISA and SEAP-activity 30 h post transfection. In some experiments, we added coverslips to the wells to analyse subcellular localization or FRET signals via confocal microscopy.

### FACS-FRET and Confocal Microscopy

**Figure 1:**
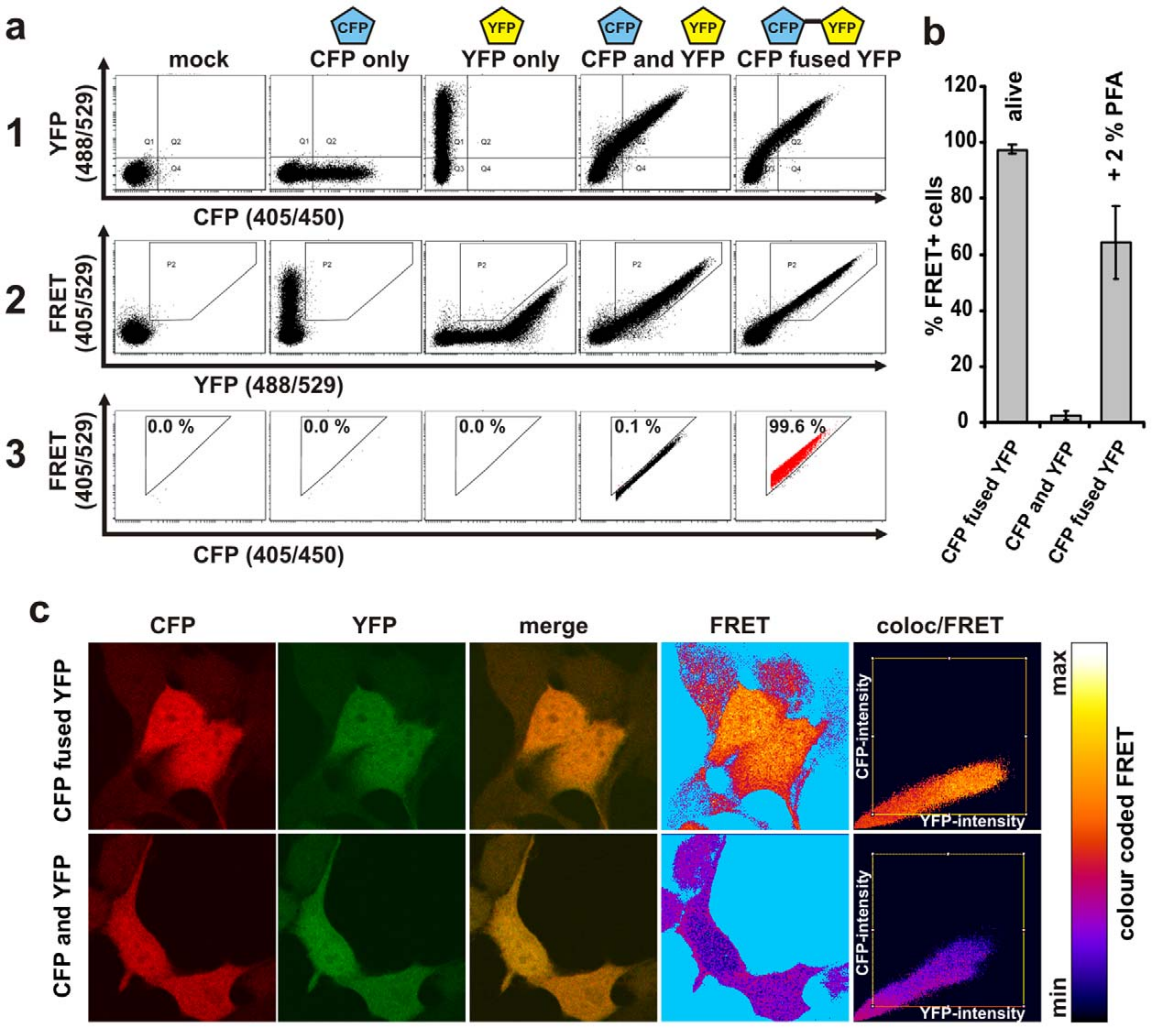
Setup of FRET-measurements by flow cytometry and microscopy. (a) The experimental setup and gating strategy to measure FRET by FACS. Living 293T cells transfected with the controls CFPonly, YFPonly, CFP and YFP as well as the CFP-YFP fusion proteins were analysed on a FACS Aria flow cytometer. Double positive cells were gated (panel 1) and false positive FRET signals resulting from YFP excitation by the 405 nm laser were excluded (panel 2). The remaining cells were evaluated for FRET by adjusting a gate defining to cells which are cotransfected with CFP and YFP only and should thus be FRET-negative (panel 3). (b) Living 293T cells and cells from the same transfections were treated with 2% PFA and analysed for FRET as depicted in (a). Shown are mean values +/− standard deviation from seven independent transfections. (c) 293T cells were grown on cover slips and cotransfected with CFP and YFP or the CFP-YFP fusion protein and mounted on microscope slides. Confocal images were taken and analysed for FRET using the “FRET and colocalization analyzer” ImageJ plug-in (7). “FRET”-images give the calculated amount of FRET for each pixel in the merged images. The ImageJ plug-in colour codes the relative FRET efficiency which is indicated by the displayed colour bar. Furthermore the “coloc/FRET”-plots display pixel colocalization as well as colour coded FRET efficiency in a 2D plot. CFP is shown in red and YFP in green.

FACS-FRET measurements were performed using a FACSAria (BD Bioscience) equipped with 405 nm, 488 nm and 633 nm lasers. To measure ECFP and FRET cells were excited with the 405 nm laser and fluorescence was collected in the ECFP channel with a standard 450/40 filter, while the FRET-signal was measured with a 529/24 filter (Semrock). To measure EYFP, cells were excited with the 488 nm laser while emission was also taken with a 529/24 filter (Semrock). For each sample, we evaluated a minimum of one thousand CFP/YFP positive cells that fell within the background adjusted gate (Fig. 1a, panel 2). To analyse subcellular localization or FRET via confocal microscopy, transfected cells grown on coverslips were mounted on microscope slides using mowiol mounting solution (2.4 g polyvinylalcohol, 6 g Glycerin, 18 ml PBS) and imaged with a Zeiss LSM510 Meta. Confocal FRET analysis were performed as described in the “FRET and colocalization analyzer – Users guide”[7].

### FACS-Analysis of Cell Surface Receptor Modulation

Jurkat cells were maintained in RPMI with standard supplements and electroporated using the Microporator-MP100 (PeqLab) device as recommended by the manufacturer. Briefly, 2×10^6^ cells per electroporation were washed twice with PBS and resuspended in 100 µl R-buffer containing 5 µg Plasmid-DNA. Microporator parameters were set to pulse voltage 1300, pulse width 20 ms and number of pulses 2. Electroporated cells were cultivated in 2 ml RPMI with standard supplements. 24 h later cells were analysed via FACS for expression of CD4, MHCI and CD3 as described previously [18].

### Vpu-CD317 Co-Immunoprecipitation Experiments

For co-immunoprecipitation, 9×10^5^ 293T cells were transfected with pCMV-FLAG-CD317 (0.1 µg) and pEYFP-vpu (1 µg) or pCG-vpu (1 µg) expression plasmids as indicated. Cells were lysed by digitonin lysis buffer (140 mM NaCl, 10 mM Tris/HCl, pH 7.4, 1 mM EDTA, protease inhibitor mixture (Roche), 1% (w/v) digitonin (Calbiochem) in order to solubilize transmembrane proteins. Cleared lysates were incubated with anti-Vpu serum [20] for 90 min on ice. Immune complexes were recovered on protein G-Sepharose for 60 min, washed four times in wash buffer (lysis buffer with 0.1% digitonin), separated in 15% SDS-PAA gel, and probed in Western blot with M2-anti-FLAG-HRP antibodies (Sigma).

### Library Screening for Potential Interaction Partners

293T or Hela cells were transfected as described above or electroporated using the Microporator MP100 device (PeqLab) as recommended by the manufacturer. For electroporation, 1 µg total DNA (0.3 µg YFP construct and 0.7 µg CFP construct) was mixed with 500,000 cells. Electroporations were carried out in 100 µl tips using the following conditions: 1200 V, 20 ms, 2 pulses for 293T cells and 985 V, 35 ms, 2 pulses for Hela cells. One day post transfection/electroporation FRET-positive cells were sorted in PBS +1% FCS and pelleted. Plasmids were isolated via the QIAamp DNA Micro Kit using the protocol for cultured cells as provided by the manufacturer or the QIAprep Spin plasmid kit (Qiagen) following the protocol “Isolation of plasmid DNA from mammalian cells with QIAprep”, which had better yields. Total recovered DNA was transformed into One Shot TOP10 cells (Invitrogen) and plated on a kanamycin containing agar plate. Colonies were picked and plasmids isolated by standard miniprep following restriction analysis of the insert.

### Statistical Analyses

Statistical analyses were performed using the Graph Pad Prism Version 5.0 software package. For all calculations we used the two-tailed unpaired Students-t-test and the Mann-Whitney test, which yielded the same results.

## Results

### Measurement of FRET by FACS

To establish an assay to measure FRET signals by FACS we first analysed 293T cells expressing the CFP and YFP controls either individually, in combination or as a fusion protein (Fig. 1a). We gated on living cells according to forward and sideward scatter (FSC/SSC) and adjusted photomultiplier tube (PMT) voltages and compensation for CFP and YFP to specifically assess FRET in double positive cells (Fig. 1a, panel 1). Importantly, when excited at 405 nm, YFP exhibited some emission in the FRET-channel. Therefore, we introduced an additional gate to exclude cells from further analysis that exert a false-positive signal in the FRET-channel due to YFP only being excited at 405 nm (Fig. 1a, panel 2). Subsequently, we plotted FRET versus CFP and introduced a triangular gate to determine the amount of FRET-positive cells. This triangle was adjusted to cells which were cotransfected with CFP and YFP only and thus are FRET-negative (Fig. 1a, panel 3). This gating strategy directly visualizes the sensitized acceptor emission arising from excitation of the CFP donor at 405 nm. Strikingly, only 0.1% of cells cotransfected with CFP and YFP scored FRET+ compared to 99.6% when cells were transfected with a CFP-YFP fusion protein (Fig. 1a, panel 3). Thus, this experimental can distinguish real FRET signals from cross-talk artefacts.

### Validation of FRET by Fluorescence Microscopy

For most FRET measurements via fluorescence microscopy samples are fixed and mounted on cover slides. Therefore, we evaluated the consequence of fixation with 2% paraformaldehyde (PFA) on the FRET efficiency in our FACS assay. The amount of FRET+ cells was reduced from 97.6% (+/− 1.1% SD) in the living cell population transfected with the CFP-YFP fusion compared to 65.1% (+/−10.7% SD) when cells were fixed (Fig. 1b). This demonstrates that fixation attenuates FRET efficiency and highlights the importance of assessing FRET measurements in living cells. To confirm the results of our FACS-based FRET assay in an independent setting, we performed confocal laser scanning microscopy on our positive (CFP-YFP fusion) and negative (CFP and YFP cotransfected) controls. Confocal images were analysed with the “FRET and colocalization analyzer” ImageJ plug-in [7]. Cells transfected with CFP and YFP together or the CFP-YFP fusion alone showed intense and indistinguishable expression of both chromophores in virtually all compartments of the cells (Fig. 1c). In contrast to this, only cells that expressed the CFP-YFP fusion displayed a high amount of colour-coded FRET (Fig. 1c), confirming the results obtained with our FACS-based FRET assay (Fig. 1a and b).

### Analysis of Protein Interactions in Living Cells

**Figure 2:**
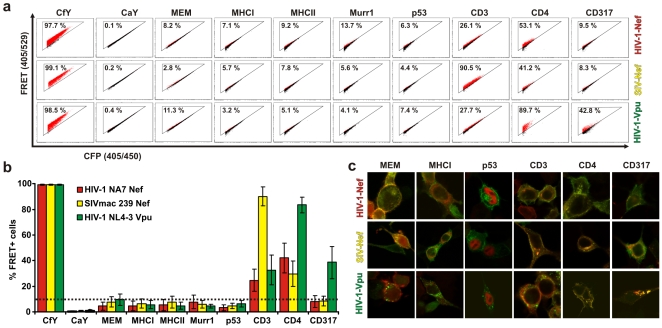
Analyses of protein interactions by FRET. (a) Representative primary FACS-plots showing the amount of FRET+ cells in living 293T cells cotransfected with the indicated CFP and YFP fusion proteins. Numbers give total percentages of cells within the FRET gate (compare Fig. 1a panel 3). (b) Mean values and standard deviations (SD) for the total amount of FRET+ cells from a minimum of eight independent experiments that were analysed as depicted in (a). The dotted line gives the maximum background FRET-signal that was obtained when cells were cotransfected with the MEM-CFP control and the YFP fusion proteins. Abbreviations: CfY, CFP fused YFP; CaY, CFPonly and YFP-fusion. (c) Merged images from confocal pictures of 293T cells that were cotransfected with the Nef/Vpu-YFP fusion proteins (shown in green) and the indicated CFP-fusions (shown in red). Regions in which both fusions colocalize appear yellow.

Next we wanted to exploit the methodology to assess the binding of HIV-1 accessory proteins engaged in a variety of interactions with cellular factors [21]. Lentiviral Nef and Vpu proteins are important for efficient viral replication and AIDS progression *in vivo* [15]. While it has been established that Nef and Vpu manipulate the surface expression of different cellular molecules, e.g. the primary viral receptor CD4 (Nef and Vpu) or MHC-I (Nef), it is a matter of intense debate whether they are modulated by direct binding to the receptors or through indirect mechanisms [15], [21], [22], [23], [24]. To address this controversial issue, we amplified genes encoding for CD4, CD3, MHC-I and II, p53 and CD317, all of which have been reported to be manipulated by Nef and/or Vpu [15], [22], [23], [24], [25] and ligated them into the pECFP-C1 fusion protein vector (Fig. S1). The well characterized human and simian immunodeficiency virus *nef* (HIV-1 NA7, SIVmac 239) [18], [22], [26] and *vpu* (NL4-3) [17], [23], [27] genes were amplified and inserted into the pEYFP-N1 vector (Fig. S1), thereby fusing EYFP to the respective C-terminus. To confirm that the viral fusions are functional, we transfected Jurkat T-cells and measured modulation of CD4, CD3 and MHC-I by Nef and Vpu (Fig. S2). Furthermore we analysed the subcellular localization of the viral as well as the cellular fusion proteins (Fig. S3). These analyses showed that the Nef and Vpu fusions are functional and exhibit the expected subcellular localization. Then we cotransfected the cellular factors together with the Nef (HIV-1 NA7, SIVmac 239) or Vpu (NL4-3) fusion proteins in 293T cells and performed FACS-based FRET on living cells (Fig. 2a and b). Nef is predominantly targeted to the cell membrane, while Vpu is located in the endoplasmic reticulum, the *trans*-Golgi-network and the plasma membrane (Fig. S3) [15], [22], [23], [24]. To address the concern that high fluorophore concentration in a specific cellular compartment (e.g. the membranes) might result in false positive FRET signals, we cotransfected the Nef- and Vpu-YFP fusions together with the MEM-CFP control. MEM-CFP is N-terminally palmitoylated and preferentially stains cellular membranes (Fig. S3) [28]. This additional negative control gave background FRET signals ranging from 4.5% (+/−3.1% SD; HIV-1 NA7 Nef) to 7.8% (+/−3.9% SD; SIVmac 239 Nef) and 9.5% (+/−4.5% SD; HIV-1 NL4-3 Vpu) respectively.

We did not measure a significant FRET signal when Nef or Vpu were expressed together with major-histocompatibility-complex class I or II (MHC-I or II), the tumor suppressor p53 or Murr-I, proposed to restrict HIV-1 replication in resting T-cells [29] (Fig. 2a and b). In contrast, expression of SIVmac 239 Nef together with the CD3ζ-chain resulted in 90.2% (+/−7.4% SD) FRET+ cells, indicating that both proteins interact (Fig. 2a and b). Only SIV Nefs, but not HIV-1 Nef or Vpu have been reported to down-modulate CD3 [15], [22], [26]. Thus, it has to be noted that HIV-1 NA7 Nef 24.6% (+/−8.6% SD) and NL4-3 Vpu 32.7% (+/−11.6% SD) also interacted with CD3ζ, albeit less efficiently than SIVmac 239. This result might explain previous conflicting reports concerning the ability of HIV-1 Nef to bind to CD3ζ [30], [31].

HIV-1 Nef and Vpu as well as SIV Nef have been reported to interfere with the expression of the primary viral receptor CD4 by direct interactions [15], [23], [32]. In agreement with these previous reports, we confirm binding of HIV-1 NA7 Nef 42.1% (+/−11.7% SD), SIVmac 239 Nef 29.8% (+/−9.8% SD) and HIV-1 NL 4-3 Vpu 83.6 (+/−6.2% SD) to CD4. Finally, we investigated the potential interaction of the three viral proteins with CD317. It has been shown that CD317 inhibits HIV-1 release from infected cells and that this restriction is counteracted by Vpu [17]. Recent reports suggest that Vpu antagonizes CD317 via a beta-TRCP dependent pathway involving binding of CD317 to Vpu [33], [34]. Using our FACS-based FRET assay we show that coexpression of CD317 and Vpu results in 39.0% (+/−18.1% SD) FRET+ cells, strongly suggesting that both directly interact in living cells. This interaction is specific, since Nef from HIV-1 NA7 7.9% (+/−4.6% SD) and SIVmac 239 8.4% (+/−% 3.8 SD) showed only background FRET signals (defined by the MEM-control) in combination with CD317 (Fig. 2a and b). All data were derived from at least eight independent experiments and the reported differences are statistically highly significant compared to the values obtained for the MEM-control (p<0.0001 for all cases using two-tailed unpaired Students-t-test and Mann-Whitney-test). Notably, we observed marked colocalization of some of the viral proteins with cellular factors despite lack of a significant FRET signal (Fig. 2c and Fig. S3; e.g. the MEM-control with NA7 and mac 239 Nef). This further demonstrates that our assay is sensitive to discriminate between mere co-localization in a subcellular compartment and real FRET signals. In sum, these results highlight the strength of our approach to characterize a variety of protein interactions in living cells not only qualitatively, but also in a quantitative manner.

### Mapping of Interacting Domains by FACS-FRET

**Figure 3:**
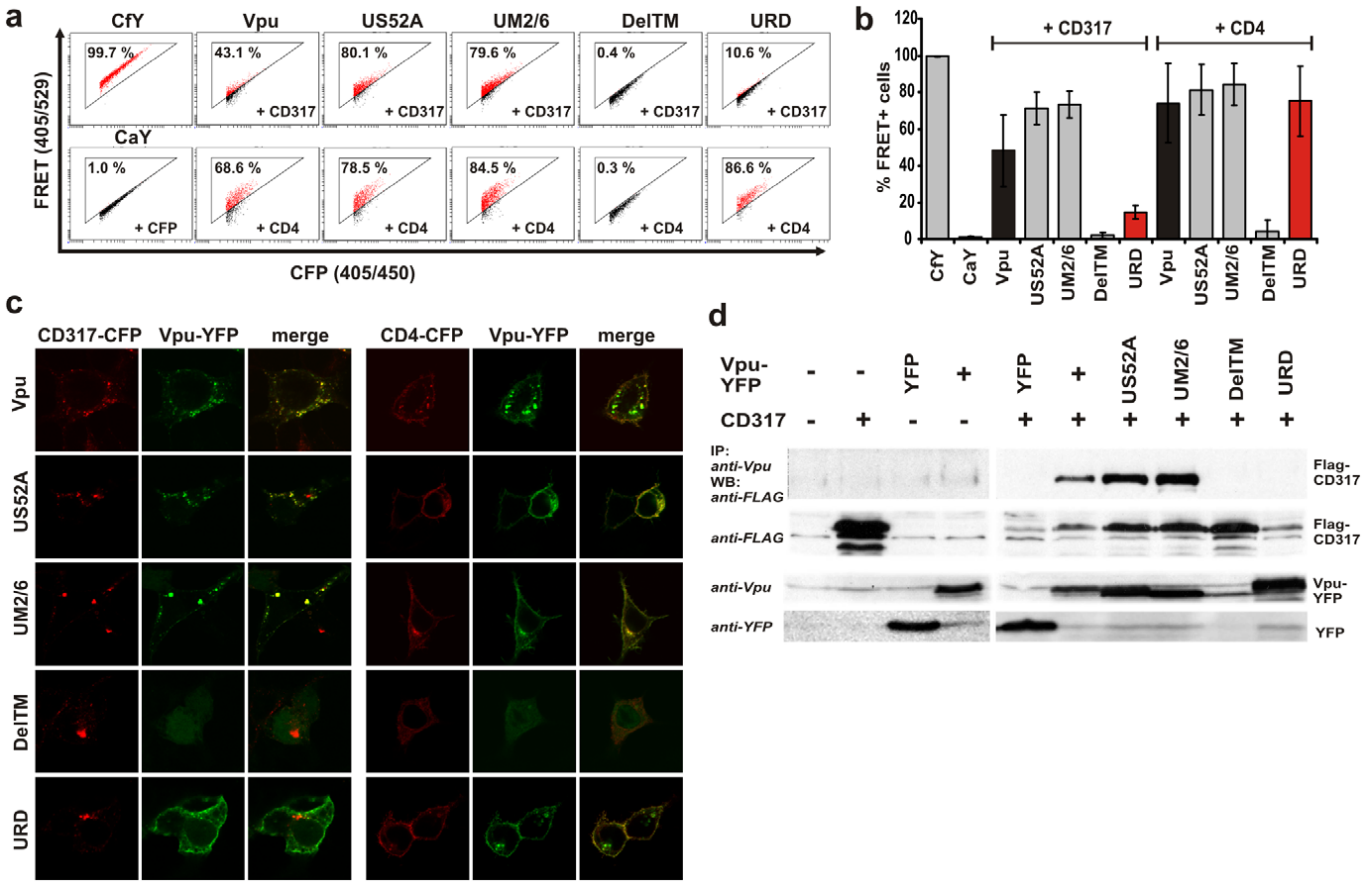
Vpu interacts with CD317 via its transmembrane region. (a) Representative primary FACS-plots showing the amount of FRET+ cells in living 293T cells cotransfected with the indicated CD4 or CD317-CFP and Vpu-YFP fusion proteins. (b) Mean values and standard deviations (SD) for the total amount of FRET+ cells from six independent experiments that were analysed as depicted in (a). (c) Confocal images of 293T cells that were cotransfected with the Vpu-YFP fusion proteins (shown in green) and either CD317-CFP or CD4-CFP (shown in red). (d) 293T cells were transfected with the indicated Vpu-YFP fusions and a FLAG-tagged CD317. Vpu immune complexes were isolated from cell detergent extracts by immunoprecipitation with anti-Vpu rabbit serum and analysed for the presence of CD317 by Western blot with anti-FLAG.

To investigate whether our assay allows to map interacting domains within proteins, we analysed a variety of previously described Vpu mutants [27] for their capacity to bind CD4 and CD317 (Fig. 3). As demonstrated in Figure 2, expression of NL4-3 Vpu together with CD4 or CD317 resulted in a strong FRET-signal (Fig. 3a and b). Mutation of one (US52A) or both (UM2/6) of the serine residues in Vpu that have been shown to be phosphorylated by casein-kinase-2 (CK-2) [23], [35] did not reduce the frequency of FRET+ cells. In contrast, deletion of the transmembrane (TM) region in Vpu (DelTM) resulted in a complete loss of FRET. Vpu RD (URD) is a mutant which contains a randomized amino acid sequence in the membrane spanning region and is therefore impaired in the enhancement of viral particle release, but not in its ability to degrade CD4 [27]. Strikingly, URD expressed together with CD317 showed a strongly diminished FRET signal 14.5% (+/−3.8% SD). To the contrary, coexpression of URD and CD4 resulted in a wild-type like FRET signal 75.1% (+/−18.7% SD). It has been reported that the degree of Vpu colocalization with CD317 correlates with functional repression of the restricting activity on HIV-1 release [36]. Therefore, we assessed the subcellular localization of the Vpu-YFP fusions together with CD317-CFP and CD4-CFP (Fig. 3c). In agreement with the results obtained by FRET, Vpu wild-type as well as US52A and UM2/6 colocalized with CD317 and CD4. DelTM lost its ability to localize to membranes and was diffusely distributed inside the cells. In contrast, URD colocalized with CD4, but not with CD317 (Fig. 3c). To biochemically confirm the results of our FRET experiments we performed co-immunoprecipitation experiments with CD317 and the various Vpu mutants. As expected, CD317 co-immunoprecipitated with Vpu wild-type and the phosphorylation mutants US52A und UM2/6, but not with the DelTM or URD mutants (Fig. 3d and Fig. S4). In summary, these results support functional studies [23], [37] suggesting that Vpu binds CD4 with its alpha-helical domain outside the TM region and targets it to proteasomal degradation, a function that requires phosphorylation of serine residues at positions 52 and 56 [23], [37]. Moreover, our results provide the first evidence that the Vpu/CD317 interaction is mediated by specific residues in the TM region of Vpu and strongly suggest that direct binding of Vpu to CD317 is necessary to overcome its restricting activity on HIV-1 release.

### Detection of Non-Membrane Localized Interactions by FACS-FRET

**Figure 4:**
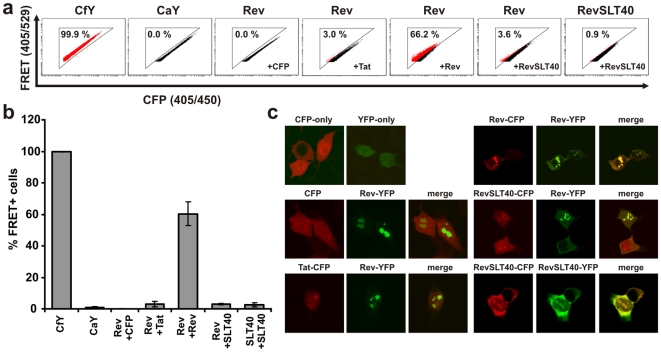
Measurement of HIV-1 Rev multimerization by FACS-FRET. (a) Representative primary FACS-plots showing the amount of FRET+ cells in living 293T cells cotransfected with the indicated CFP and YFP fusion proteins. (b) Mean values and standard deviations (SD) for the total amount of FRET+ cells from four independent experiments that were analysed as depicted in (a). (c) Confocal images of 293T cells that were cotransfected with the indicated YFP (shown in green) and CFP (shown in red) fusion proteins.

Lentiviral Nef and Vpu proteins are mainly targeted to cellular membranes and interact with other membrane associated receptors or signalling complexes. To demonstrate that our assay is also useful to investigate interactions of non-membrane bound proteins we measured multimerization of the HIV-1 Rev protein, that is important for the nuclear export of unspliced and incompletely-spliced viral mRNAs [16]. CFP/YFP fusions of HIV-1 Rev were able to mediate nuclear export of a Rev-responsive mRNA reporter construct [19] and expression of the trans-dominant Rev multimerization mutant SLT40 inhibited the biological activity of wild-type Rev as previously reported [16] (Fig. S5). Co-expression of CFP and YFP labelled Rev in 293T cells resulted in 66.2% (+/−7.5% SD) of FRET positive cells (Fig. 4a and b). As controls and to verify our previous results that the assay is sensitive to discriminate co-localization from FRET, we measured FACS-FRET between Rev, the viral transactivator Tat and the multimerization-deficient RevSLT40 mutant. As expected, we did not measure significant FRET (Fig. 4a and b), despite strong colocalization of these proteins in the cyto- and nucleoplasm, as well as in nuclear microbodies (i.e. nucleoli) (Fig. 4c). In sum, these results show the applicability of FACS-FRET to assess interactions of non-membrane localized proteins.

### FACS-Based FRET as a Tool for High-Throughput Screening (HTS)

**Figure 5:**
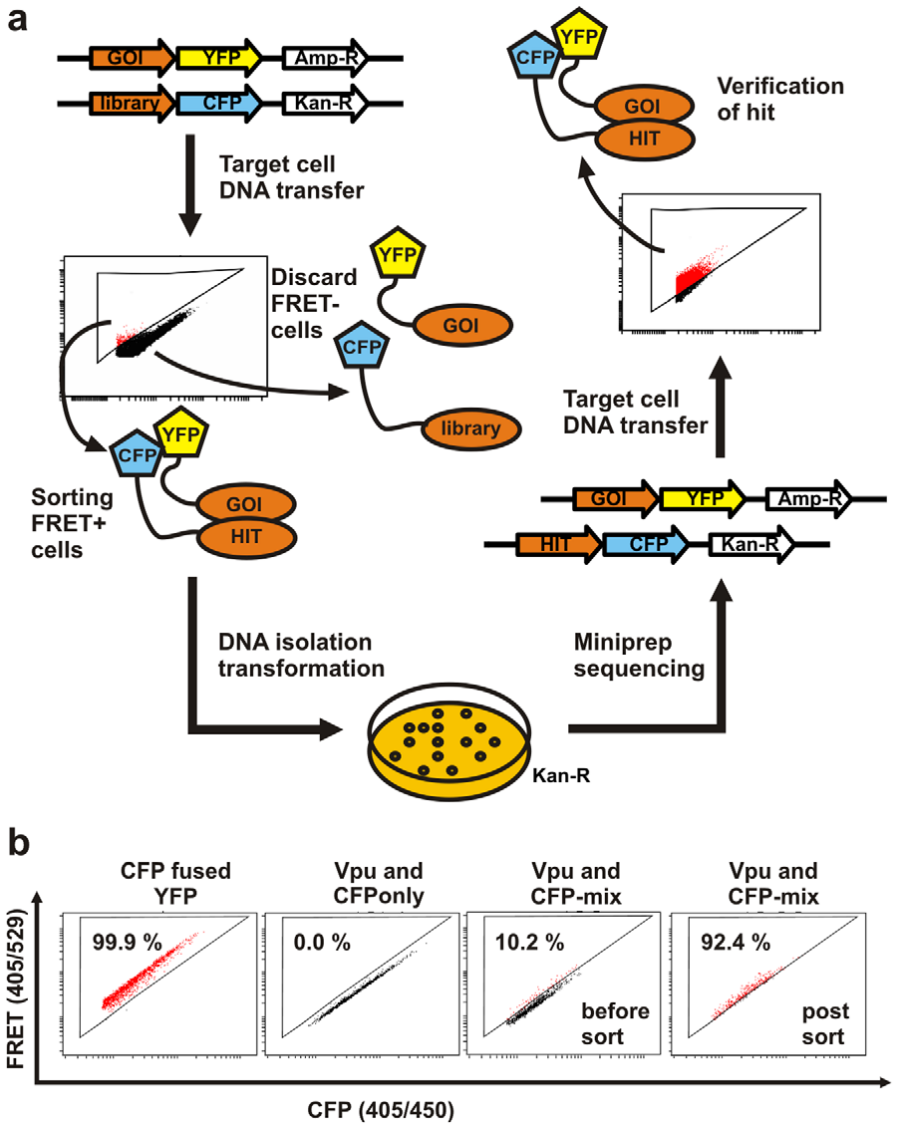
High-throughput-screening for unknown protein interactions by FACS-FRET. (a) Experimental setup to screen for unknown protein interaction with flow cytometry based FRET in high-throughput. (b) Living 293T cells were transfected with the Vpu-YFP fusion as a bait and a mixture of equal amounts of the CFP-fusion constructs that are described in Figure 2. 36 h post transfection FRET+ cells were sorted, pelleted, resuspended in PBS and reanalysed for successful purification. Abbreviation GOI, gene of interest.

Mammalian- or yeast-two-hybrid assays are the most commonly used methods to screen for protein interactions *in vivo* [1], [2]. While this technology is a powerful tool, FRET has the significant advantage that it does not require the interaction to take place within the nucleus. Therefore, we established FACS-based FRET for HTS of protein interactions from a mixture of cDNAs (Fig. 5A). First, we ligated the Vpu-YFP fusion sequences in the pCGCG-vector containing the ampicillin resistance gene [18]. Next, as proof of principle, we mixed a cDNA library containing all the cellular factors that have been analysed before (Fig. 2). Subsequently, we transfected 293T cells with the Vpu-YFP construct and the mixture of CFP-fusions and sorted FRET+ cells. Prior to sorting, 10.2% of cells transfected with the CFP-mixture and Vpu-YFP scored FRET positive (Fig. 5B). Reanalysis of the sorted cell fraction revealed that now 92.6% of cells exerted a FRET signal (Fig. 5B), validating the purification of FRET+ cells.

Next, we performed the entire procedure with the mixed virtual cDNA-library exactly as depicted in Fig. 5A. Using 293T cells and CaCl transfection we were able to reisolate plasmids from down to 5,000 sorted cells. Restriction analyses of the plasmids revealed that we isolated on average 50% to 60% of false positives together with the Vpu binding partners, probably due to the presence of multiple plasmids per cell and their amplification by the SV40 large t-antigen, which is constitutively expressed in 293T cells. While the rate of false positives in our approach seems high at first, it is acceptable in comparison to yeast-two-hybrid screens which have an estimated number of false positives ranging from 50% up to 90% [38], [39]. In sum, these results establish FACS-based FRET as a useful tool to screen for protein interaction partners from a cDNA mixture and warrant its application in future high-throughput-screens.

## Discussion

Our study describes a novel methodology to detect protein interactions in living cells by combining FRET and flow cytometry. Most importantly, compared to previous reports that measured FRET by FACS [9], [10], [11], [12], [13], we designed an approach that allows quantification and statistics, eliminates cross talk artefacts and is easy to adapt to other applications. This renders the method accessible to researchers that are not familiar with FRET or complex FACS measurements. Moreover, by employing biochemical methods (Fig. 3) we proved that our FACS-FRET results reflect bona fide physical interactions that can be detected in any cellular compartment (e.g. Nef at the plasma-membrane; Vpu, which localizes to the plasma- and ER-membrane and the *trans-*Golgi network, as well as Rev multimer-formation in the cytosol, nucleus and nucleoli). We used a standard equipped flow cytometer (FACSAria) and worked with widely distributed cells, which are easy to cultivate and to transfect (293T and HeLa cells, CaPO transfection and electroporation). Furthermore, we established cloning strategies that allow the generation of any gene of interest either as N- or C-terminal fusions with the standard FRET pair ECFP or EYFP (Fig. S1).

An unique advantage of our approach is that FRET-efficiency can easily be quantified as percentage of cells scoring FRET-positive. The FRET signal is affected by numerous variables, e.g. the chosen fluorophores, sterical orientation of the fluorophores, expression and size of the fusion protein, quantity of interacting proteins and, finally, distance between both interaction partners [3], [4]. Thus, the amount of FRET is a direct measurement of the permanent proximity of two proteins inside a cell and is a strong indicator for direct interaction. However, one has to be careful when drawing conclusions from the FRET-efficiency on the strength of an interaction. For example, our results show that HIV Nef and Vpu as well as SIV Nef interact with CD4 qualitatively (Fig. 2). Nevertheless, despite the higher FRET-efficiency of Vpu together with CD4 we can not conclude that there is more or stronger interaction, because the three dimensional architecture of the Vpu fusion is fundamentally different from that of the Nef fusions. On the other hand, the significant higher FRET-efficiency of SIV Nef with CD3 suggests a stronger binding efficiency compared to HIV-1 Nef (Fig. 2), since both proteins are characterized by a comparable structure [22]. Importantly, this result might explain conflicting reports concerning the ability of HIV-1 Nef to bind to CD3 in the past [30], [31]. Furthermore, in a setting that keeps the variety of FRET parameters constant, we could not only show that HIV-1 Vpu interacts with CD317 in living cells, but also map functional interaction domains by FACS-FRET (Fig. 3). Most interestingly VpuRD, a mutant that is defective in the enhancement of HIV-1 particle release [27], was unable to bind to CD317 but fully retained its ability to interact with CD4. In agreement with a recent report [34], this strongly suggests that interaction of Vpu with CD317 is functionally required to overcome its restricting activity on HIV-1 release.

An inherent problem of FRET measurements is the required tagging of proteins with fluorophores. For one, a tag may affect the functionality and proper localization of proteins. In addition, the spatial orientation of the proteins to be analysed might prevent the fluorophores to come into close proximity, thereby preventing emission of a FRET signal, despite ongoing protein-protein interaction. False negative FRET signals could also be the result of fusion protein interaction with endogenously expressed interaction partners, reducing the overall FRET efficiency. This could be the case for the putative interaction of Nef with MHC-I [24], which could not be confirmed by FACS-FRET (Fig. 2). In sum, negative FRET results do not allow to exclude the possibility that two proteins interact. A positive FACS-FRET signal, however, is a strong indicator for a physiological interaction *in vivo*. In addition to the demonstration of such an interaction, FACS-FRET offers a variety of down-stream applications, e.g. mapping of interaction domains (Fig. 3) or screening for interfering drugs and thus has the potential to give rise to new therapeutic treating options for human diseases.

High-throughput-screening (HTS) for protein interactions is one of the major bottlenecks in proteomics. The most frequently used method, yeast-two-hybrid analyses, is a powerful tool, but also has serious limitations. For example, post-translational modifications of proteins in yeast are different from mammalian cells and interactions have to take place in the nucleus [2]. Moreover, yeast-two-hybrid screens are technically challenging and time consuming. HTS by FACS-FRET has been demonstrated before, but only in *E.coli* and due to the specific experimental setup and the used chromophores it was only possible to perform the screen with small fragments of a protein [13]. As proof of principle, we demonstrate successful screening for full length interaction partners in living mammalian cells by FACS-FRET (Fig. 5). Our false-positive rate ranging from 50% to 60% is comparable or even better than the estimates for yeast-two-hybrid screens [38], [39], [40]. Furthermore, it is possible to render the screen more stringent by adjusting the FRET-gate on the cost of isolated cells. In our experiments we succeeded to reisolate plasmids from down to 5,000 sorted 293T cells. However, it must be noted that with such low cell numbers the total yield of plasmids was rather low ranging around 30 colonies. Thus, for a true screen it is required to sort substantially more cells. As already mentioned, the high amount of false-positives is probably due to multiple plasmids that are transfected per cell and which are subsequently reisolated along with the “true” hits. We tried to avoid this problem by reducing the amount of DNA and changing the method of DNA delivery (e.g. electroporation). However, this measures always resulted in a significant loss of recovered DNA. Current experiments in our lab focus on the improvement of the screen by (i) the evaluation of novel chromophores that might have an improved FRET-efficiency, (ii) testing of other cells and transfection methods and (iii) alternative approaches to reisolate the plasmids from the sorted cells. Nevertheless, we also currently exploit the screen as it is presented in this report to identify novel interaction partners of HIV-1 proteins from a cloned T-cell cDNA library.

Altogether, FACS-FRET has several significant advantages compared to existing methods. It allows to detect protein interactions in all cellular compartments, it is fast and quantitative, non-invasive and highly reproducible. Thus, the FACS-based FRET assay presented herein may significantly improve our prospects to define protein interactions in living mammalian cells.

## Supporting Information

Figure S1 Expression vectors for the generation of fusion proteins. Unmodified pEYFP- or pECFP-C1/N1 (Clontech) vectors were chosen for the generation of fusion proteins. C-terminal with chromophore tagged fusions can be generated in either the C1 or the N1 vector backbone by using single NheI and AgeI restriction sites. The gene of interest (GOI) is cloned in frame with the chromophore post elimination of the stop codon and introduction of the linker sequence GAGAPVAT by PCR. N-terminal with chromophore tagged fusions can be generated in the C1-backbone by XhoI and EcoRI sites or in the N1-backbone using BsrGI and NotI together with the linkers indicated.
(0.27 MB TIF)

Figure S2 Analyses of cell surface receptor modulation by Nef and Vpu fusion proteins. Jurkat cells were electroporated with pEYFP-only, pEYFP-MEM, pEYFP HIV-1 NA7 Nef, pEYFP-SIV mac239 Nef or pEYFP-NL4-3 Vpu and down-modulation of CD4, CD3 and MHC-I by the different viral proteins was measured by flow cytometry as described in the methods section. Receptor cell surface expression of pEYFP-only electroporated cells was set as 100%. Presented are means and standard deviations of two independent experiments.
(0.23 MB TIF)

Figure S3 Colocalization and subcellular localization of viral and cellular fusion-proteins. Confocal images of 293T cells that were cotransfected with the indicated YFP- and CFP-fusion proteins. The top panel shows three different cells that were transfected with the indicated YFP-fusion proteins only. The left panel shows individual cells that were transfected with the indicated CFP-fusion proteins only. YFP is shown in green and CFP is shown in red.
(2.36 MB TIF)

Figure S4 Untagged NL4-3 Vpu protein immunoprecipitates CD317. 293T cells were transfected with the pCG-NL4-3 Vpu and a FLAG-tagged CD317. Vpu complexes from cellular lysates were immunoprecipitated with a rabbit anti-Vpu serum (43) and blotted for the presence of CD317 with anti-FLAG.
(0.23 MB TIF)

Figure S5 Biological activity of HIV-1 Rev CFP/YFP fusion proteins. 293T cells were transfected with the indicated CFP/YFP fusion proteins and co-transfected with the Gag expression vector GPV-RRE (36) and a CMV-SEAP reporter construct. Released p24 was measured by ELISA and normalized to transfection efficiency by determining the levels of SEAP (secreted alkaline phosphatase). Error bars represent the SD of triplicates from one representative out of two independent experiments.
(0.19 MB TIF)
