# Supplementary figures and images for: A Flow Cytometry-Based FRET Assay to Identify and Analyse Protein-Protein Interactions in Living Cells
Source: PLoS One. 2010 Feb 22;5(2):e9344. doi: 10.1371/journal.pone.0009344 (PMC2825263; doi:10.1371/journal.pone.0009344)

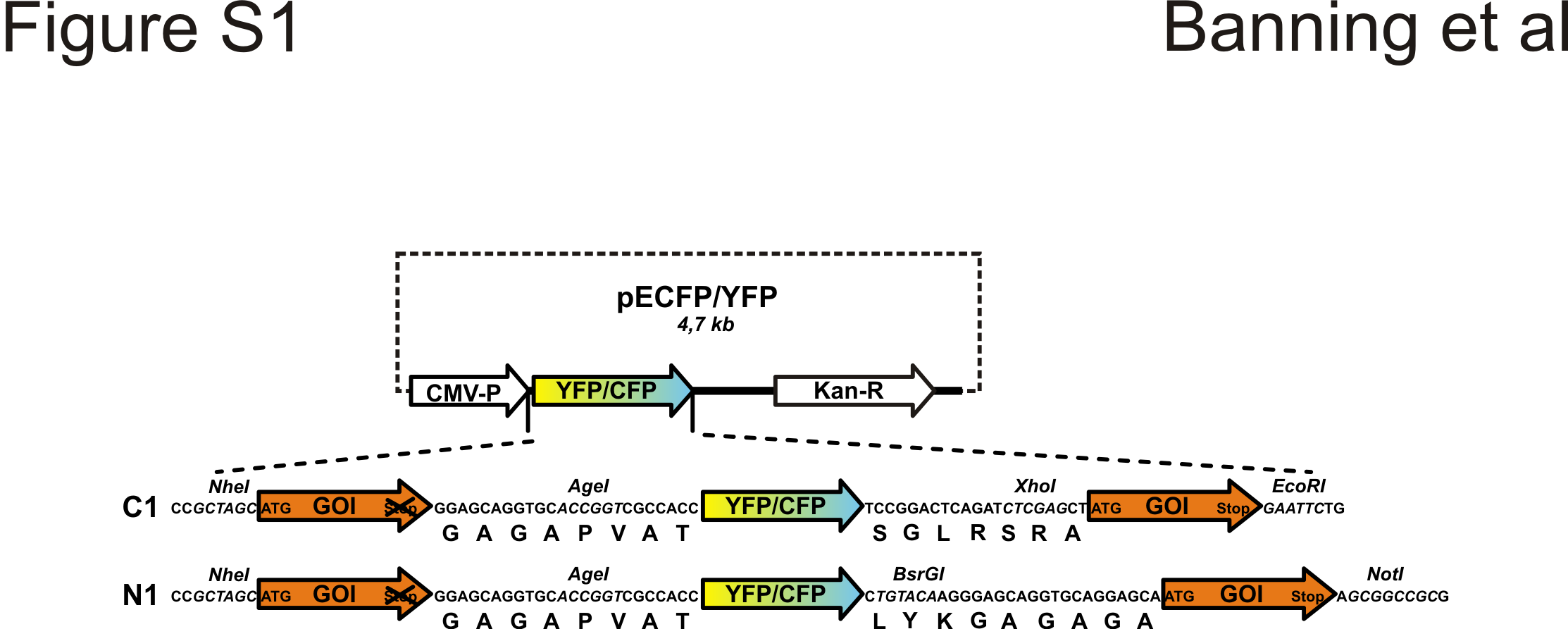

Supplement: Figure S1 — Expression vectors for the generation of fusion proteins. Unmodified pEYFP- or pECFP-C1/N1 (Clontech) vectors were chosen for the generation of fusion proteins. C-terminal with chromophore tagged fusions can be generated in either the C1 or the N1 vector backbone by using single NheI and AgeI restriction sites. The gene of interest (GOI) is cloned in frame with the chromophore post elimination of the stop codon and introduction of the linker sequence GAGAPVAT by PCR. N-terminal with chromophore tagged fusions can be generated in the C1-backbone by XhoI and EcoRI sites or in the N1-backbone using BsrGI and NotI together with the linkers indicated. (0.27 MB TIF) [file pone.0009344.s001.tif]

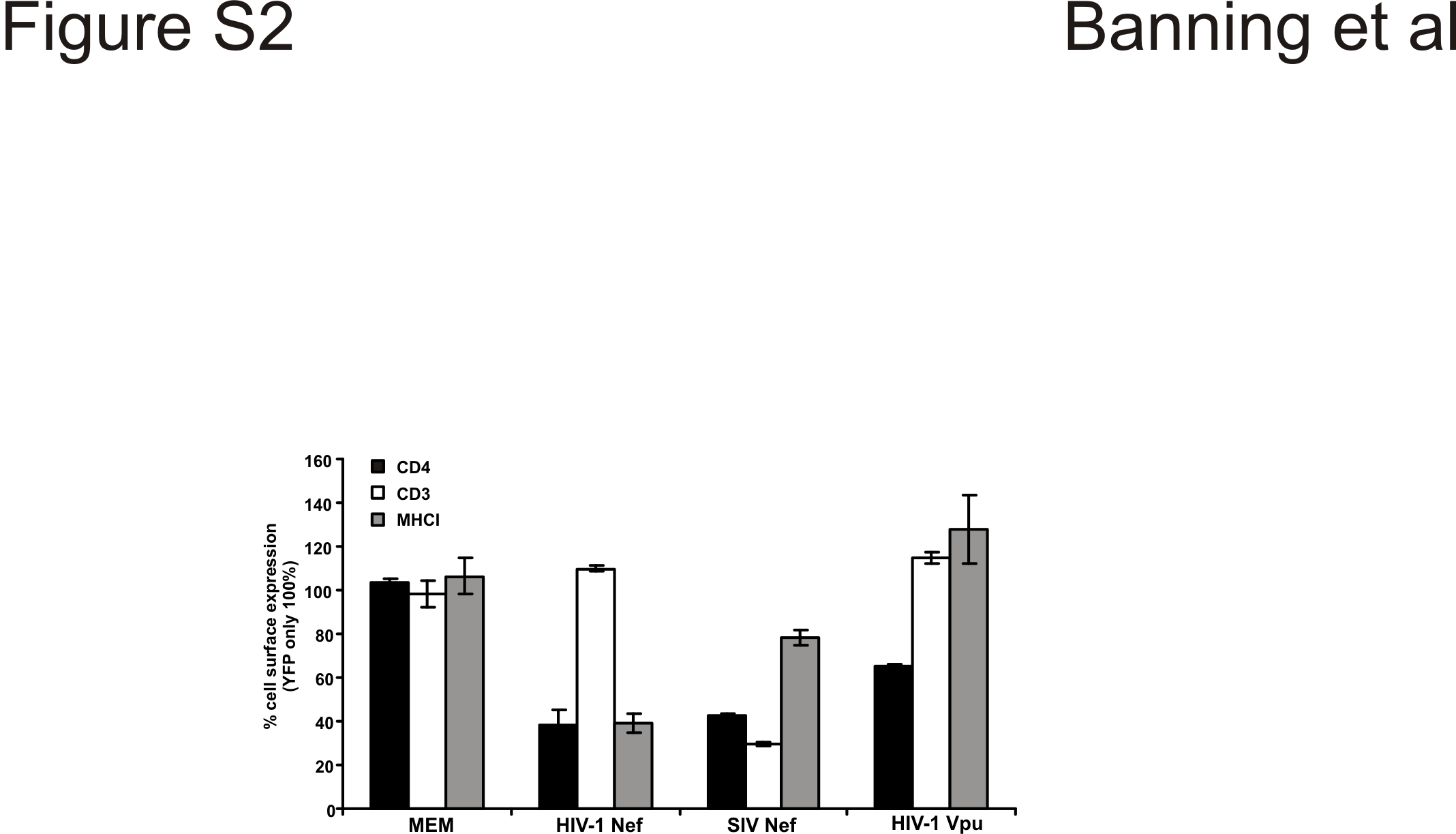

Supplement: Figure S2 — Analyses of cell surface receptor modulation by Nef and Vpu fusion proteins. Jurkat cells were electroporated with pEYFP-only, pEYFP-MEM, pEYFP HIV-1 NA7 Nef, pEYFP-SIV mac239 Nef or pEYFP-NL4-3 Vpu and down-modulation of CD4, CD3 and MHC-I by the different viral proteins was measured by flow cytometry as described in the methods section. Receptor cell surface expression of pEYFP-only electroporated cells was set as 100%. Presented are means and standard deviations of two independent experiments. (0.23 MB TIF) [file pone.0009344.s002.tif]

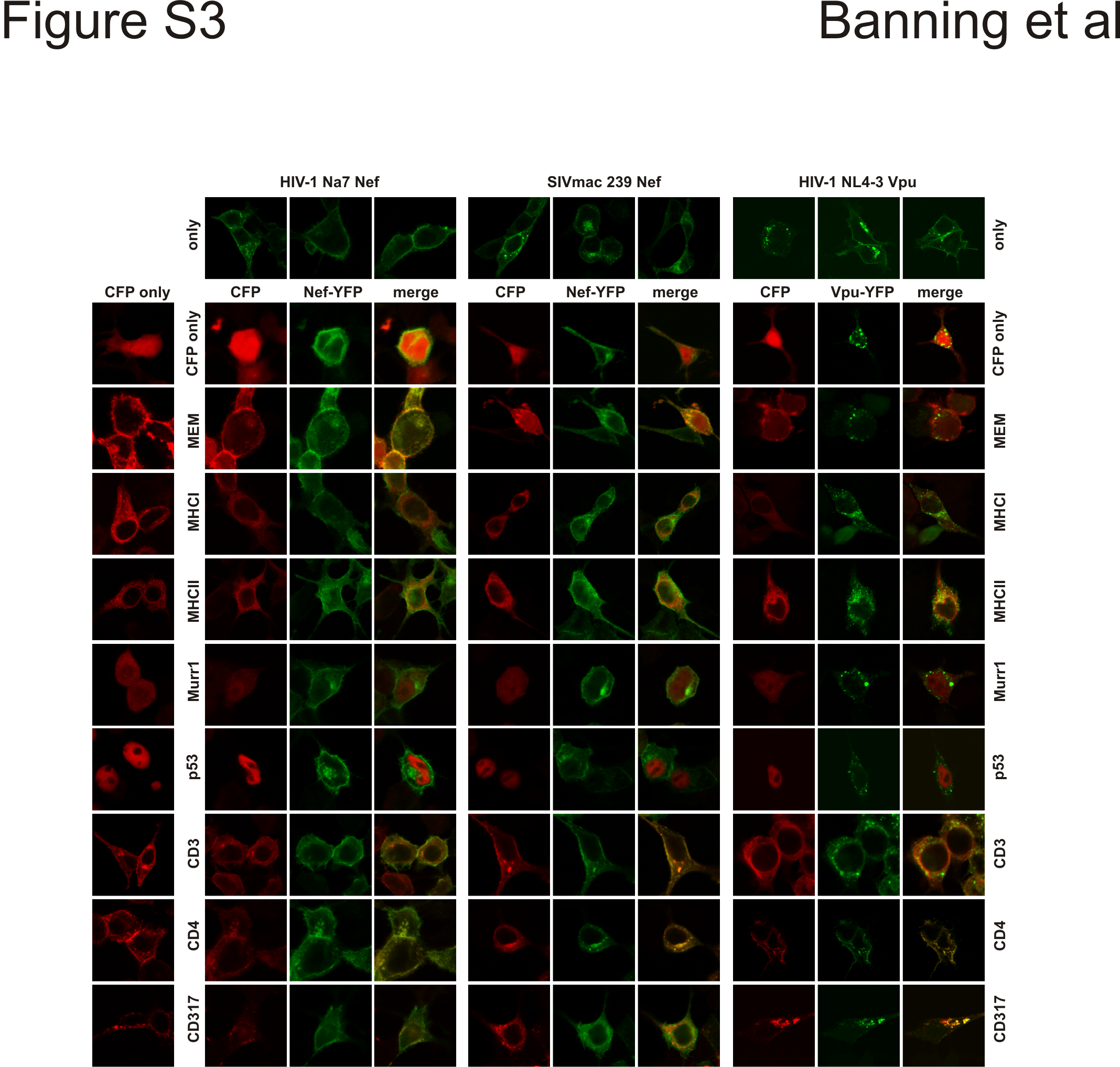

Supplement: Figure S3 — Colocalization and subcellular localization of viral and cellular fusion-proteins. Confocal images of 293T cells that were cotransfected with the indicated YFP- and CFP-fusion proteins. The top panel shows three different cells that were transfected with the indicated YFP-fusion proteins only. The left panel shows individual cells that were transfected with the indicated CFP-fusion proteins only. YFP is shown in green and CFP is shown in red. (2.36 MB TIF) [file pone.0009344.s003.tif]

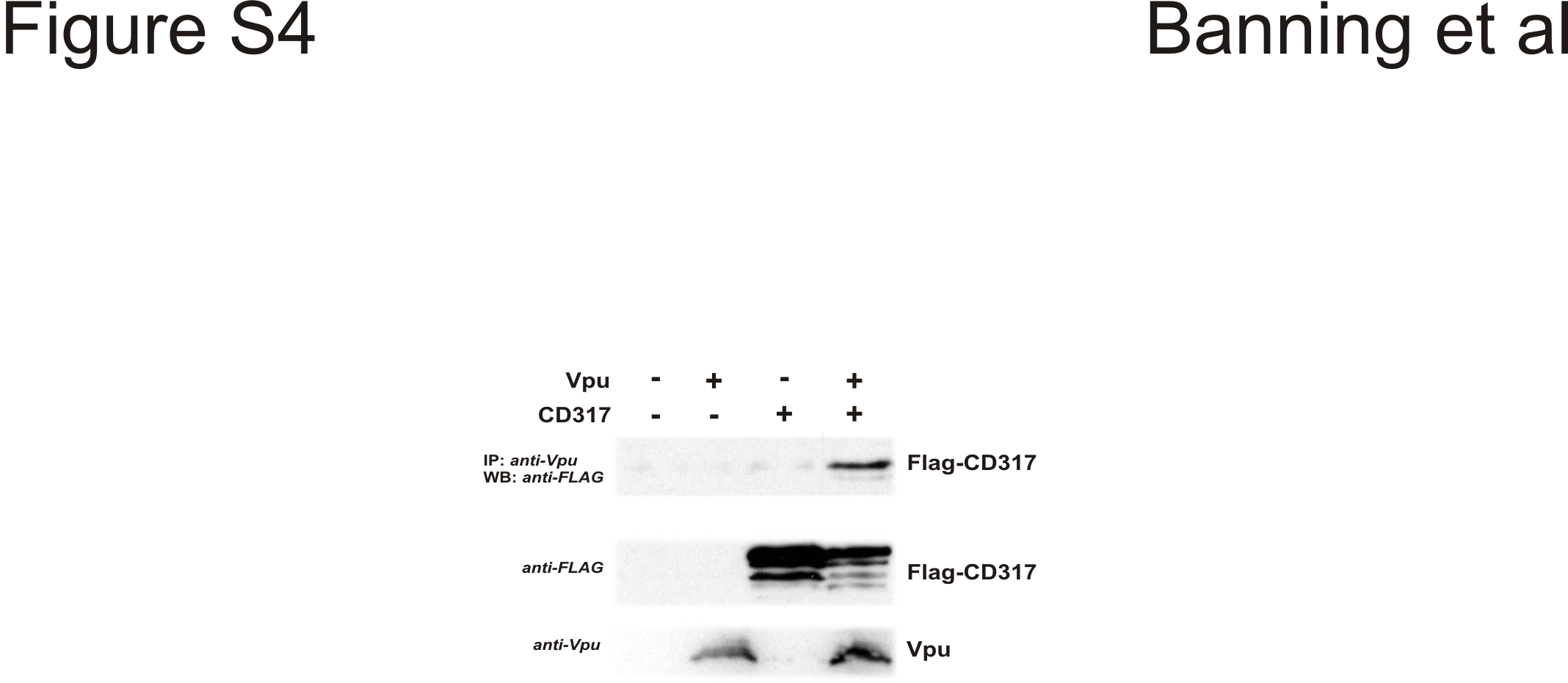

Supplement: Figure S4 — Untagged NL4-3 Vpu protein immunoprecipitates CD317. 293T cells were transfected with the pCG-NL4-3 Vpu and a FLAG-tagged CD317. Vpu complexes from cellular lysates were immunoprecipitated with a rabbit anti-Vpu serum (43) and blotted for the presence of CD317 with anti-FLAG. (0.23 MB TIF) [file pone.0009344.s004.tif]

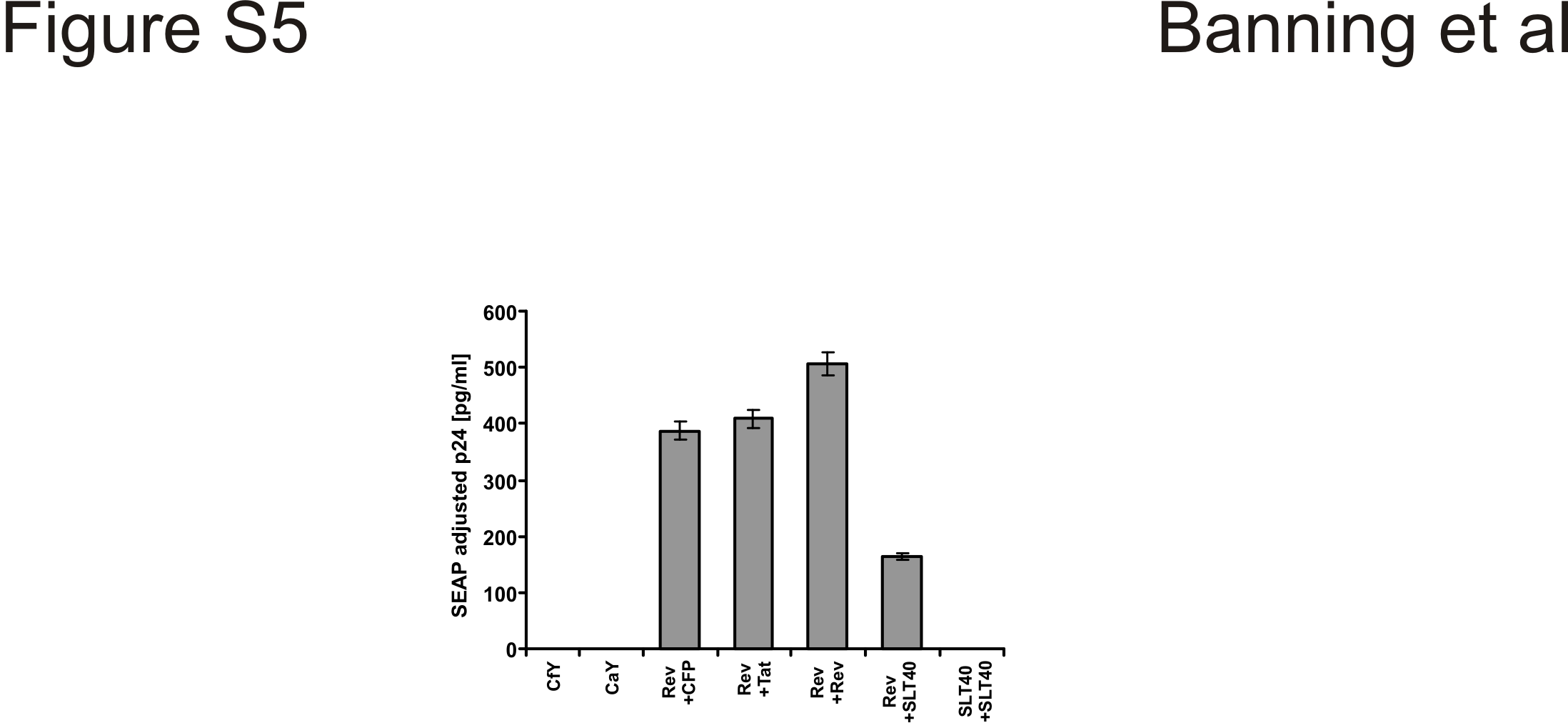

Supplement: Figure S5 — Biological activity of HIV-1 Rev CFP/YFP fusion proteins. 293T cells were transfected with the indicated CFP/YFP fusion proteins and co-transfected with the Gag expression vector GPV-RRE (36) and a CMV-SEAP reporter construct. Released p24 was measured by ELISA and normalized to transfection efficiency by determining the levels of SEAP (secreted alkaline phosphatase). Error bars represent the SD of triplicates from one representative out of two independent experiments. (0.19 MB TIF) [file pone.0009344.s005.tif]
